# Analysis of correlation between BMI and TWL% outcome following metabolic and bariatric surgery: a retrospective study using restricted cubic spline

**DOI:** 10.1186/s12893-024-02455-7

**Published:** 2024-06-07

**Authors:** Guanyang Chen, Zhehong Li, Liang Wang, Qiqige Wuyun, Qing Sang, Jing Wang, Zheng Wang, Chenxu Tian, Chengyuan Yu, Buhe Amin, Nengwei Zhang, Qing Fan

**Affiliations:** 1 Department of General Surgery, Peking University Ninth School of Clinical Medicine, Beijing, China; 2 Department of Critical Care Medicine, Peking University People’s Hospital, Beijing, China; 3 Department of General Surgery, Beijing Shijitan Hospital, Capital Medical University, Beijing, China

**Keywords:** Body mass index, Total weight loss, Restricted cubic spline, Metabolic and bariatric surgery

## Abstract

**Objective:**

This study aimed to examine the correlation between preoperative body mass index (BMI) and adequate percentage of total weight loss (TWL%) outcome and present evidence of tiered treatment for patients with obesity in different preoperative BMI.

**Methods:**

We included patients with complete follow-up data who underwent metabolic and bariatric surgery (BMS). We termed optimal clinical response as TWL% >20% at one year following MBS. To investigate dose-response association between preoperative BMI and optimal clinical response, preoperative BMI was analyzed in three ways: (1) as quartiles; (2) per 2.5 kg/m2 units (3) using RCS, with 3 knots as recommended.

**Results:**

A total of 291 patients with obesity were included in our study. The corresponding quartile odds ratios associated with optimal clinical response and adjusted for potential confounders were 1.00 (reference), 1.434 [95% confidence interval (95%CI)   =  0.589–3.495], 4.926 (95%CI   =  1.538–15.772), and 2.084 (95%CI   =  0.941–1.005), respectively. RCS analysis showed a non-linear inverted U-shaped association between preoperative BMI and optimal clinical response (Nonlinear P   =  0.009). In spline analysis, when preoperative BMI was no less than 42.9 kg/m^2^, the possibility of optimal clinical response raised as preoperative BMI increased. When preoperative BMI was greater than 42.9 kg/m^2^, the possibility of optimal clinical response had a tendency to decline as preoperative BMI increased.

**Conclusion:**

Our research indicated the non-linear inverted U-shaped correlation between preoperative BMI and adequate weight loss. Setting a preoperative BMI threshold of 42.9 is critical to predicting optimal clinical outcomes.

**Supplementary Information:**

The online version contains supplementary material available at 10.1186/s12893-024-02455-7.

## Introduction

Over the past several decades, the global incidence of patients with obesity has rised dramatically and shows no signs of slowing [1–3]. A number of diseases linked to increased mortality is more likely to be caused by obesity. These diseases include type 2 diabetes mellitus (T2DM) [4, 5], cardiovascular disease [2], depression [6], and cancers [7, 8]. Diet, exercise, cognitive behavioral therapy, medications, and metabolic and bariatric surgery (MBS) are the current therapeutic alternatives for treating obesity [9–11]. Among them, MBS is the most effective treatment for patients with obesity [12]. Laparoscopic Roux-en-Y gastric bypass (LRYGB) and laparoscopic sleeve gastrectomy (LSG) have been the most commonly performed obesity surgeries in the last decade [13, 14]. While most patients with obesity achieve optimal clinical response, total weight loss (TWL%), after MBS, 30-40% still struggle to achieve this goal [15–17]. Suboptimal clinical response may be the result of a combination of variables, including the level of the surgical team and the patient-level characteristics [15, 18].

The body mass index (BMI) is the most often used anthropometric statistic to define obesity [19]. However, the association between preoperative BMI and optimal clinical response remains contentious. Some studies have proposed that higher preoperative BMI is associated with lower weight loss [20, 21]. However, some studies have suggested that a larger preoperative BMI is a negative predictor of postoperative weight loss [22, 23]. This controversial predictive result suggests that traditional linear analyses used to assess the relationship between baseline BMI and weight loss outcomes are not applicable.

Restricted cubic spline (RCS) is essential for examining dose-response interactions between continuous variables and outcomes [24, 25]. This study aimed to examine the association between preoperative BMI and optimal clinical response using logistic regression and RCS. Provide personalized support by analyzing the preoperative BMI of patients with obesity, thereby increasing the likelihood of achieving optimal clinical outcomes.

## Methods

### Study participants

In our study, we collected data of patients with complete follow-up information who underwent MBS at Beijing Shijitan Hospital from April 2012 to October 2019. The detailed inclusion and exclusion criteria based on the Chinese Guidelines for Surgical Treatment of Obesity and Type 2 Diabetes (2019 Edition and 2014 Edition) have been described in in our previous studies [26]. We termed optimal clinical response as TWL%>20% at one year following MBS. Then, patients were divided into two groups: the optimal clinical response group (TWL%>20%) and the suboptimal clinical response group (TWL%≤20%). The Institutional Review Board (IRB) of Beijing Shijitan Hospital approved this study (Approval No. sjtkyll-lx-2019-58). The study protocol conformed to the ethical guidelines of the 1975 Declaration of Helsinki. Written informed consent was obtained from each participant.

### Definitions

1. Diagnostic Criteria for Hypertension: Hypertension was defined as systolic ≥ 140 mmHg and/or diastolic ≥ 90 mmHg according to the Chinese Hypertension Prevention Guide (2010 Revised Edition).
2. Diagnostic Criteria for T2DM: According to WHO criteria(1999): Trough glucose oxidase method, FPG 7.0 mmol/L and/or 2hPG11.1 mmol/L were considered as diabetes.
3. Diagnostic Criteria for Hyperlipidemia: Abnormal blood lipids (TC ≥ 5.18mmol/L or TG ≥ 1.70mmol/L or HDL-C < 1.04mmol/L or LDL-C ≥ 3.37mmol/L) were diagnosed as hyperlipidemia based on the criteria of “Chinese Guidelines on Prevention and Treatment of Dyslipidemia in Adults”.
4. Diagnostic Criteria for Hyperuricemia: The diagnostic criteria for HUA are that under a normal purine diet, two fasting serum uric acid (SUA) levels on different days are ≥ 420 mmol/L in men and ≥360 mmol/L in women, based on the criteria of the “Chinese Multidisciplinary Expert Consensus on the Diagnosis and Treatment of Hyperuricemia and Related Diseases”.
5. BMI   =  weight in kg/(height in m)^2^;
6. TWL% = (starting weight - current weight / starting weight) × 100%.
7. The procedures for MBS (LSG and LRYGB) are described and postoperative follow-up were described in our previous studies [26, 27].

### Grouping and models

To investigate dose-response associations between preoperative BMI and optimal clinical response, preoperative BMI was analyzed in three ways: (1) as quartiles [quartile 1 (27.50–33.15 kg/m^2^, Q1), quartile 2 (33.15–38.19 kg/m^2^, Q2), quartile 3 (38.19–43.85 kg/m^2^, Q3), and quartile 4 (> 43.85 kg/m^2^, Q4)]; (2) per 2.5 kg/m^2^ units (modeled as discrete variables as follows: 27.5  ≤  BMI < 30.0, 30.0 ≤ BMI < 32.5, 32.5  ≤  BMI < 35.0, 35.0  ≤  BMI < 37.5, 37.5  ≤  BMI < 40.0, 40.0  ≤  BMI < 42.5, 42.5  ≤  BMI < 45.0, 45.0  ≤  BMI < 47.5, 47.5  ≤  BMI < 50.0, and 50.0  ≤  BMI) (3) using RCS, with 3 knots as recommended. Initial models (Model 1) estimated odds ratios (ORs) of TWL% outcomes without adjustment. Model 2 adjusted for potential confounders (univariate logistic regression analysis significant factors).

Firstly, we divided patients with obesity into four groups according to preoperative BMI quartiles (Q1, Q2, Q3, and Q4) and ten groups according to per 2.5 kg/m^2^ units. Then, we examined the association between preoperative BMI and TWL% outcomes using the logistic regression analysis in both models 1 and 2 according to groups of quartiles and per 2.5 kg/m^2^ units, respectively. Finally, RCS curves based on logistic regression with three knots at the 10th, 50th, 90th percentiles were used to evaluate the relationship between preoperative BMI and optimal clinical response. The RCS model was then adjusted based on variables that were statistically significant in the univariate logistic regression analysis. Subgroup RCS analysis was performed based on surgery. This model was used to examine nonlinear relationships between variables. For a dose-response relationship between variables, P overall < 0.05 indicates a relationship, whereas P overall > 0.05 indicates no association. A nonlinear dose-response connection is shown by a P nonlinear < 0.05, while a P nonlinear > 0.05 indicates no such relationship.

### Statistical analyses

Categorical variables were expressed as frequency and percentages and continuous variables as mean ± standard deviation (SD). Differences in continuous variables between groups were compared by the Student t-test and one-way ANOVA for comparing more than two groups. Pearson’s chi-square test or Fisher’s exact test was used for categorical variables. Dose-response analyses using RCS functions, logistic regression analysis were conducted to evaluate the relationship between preoperative BMI and TWL% outcomes. All data were processed in the R version 4.2.1 software.

## Results

### Characteristics and groups of participants

A total of 291 patients with obesity were included in our study, and Table 1 showed the characteristics of the participants grouped by TWL% outcomes (the optimal clinical response group and the suboptimal clinical response group). Moreover, Supplementary Table S1 showed the participants’ characteristics grouped by preoperative BMI quartiles (Q1, Q2, Q3, and Q4). The median preoperative BMI across low-to-high quartiles of distribution were 30.35, 36.20, 40.40, and 48.56 kg/m^2^, respectively. Univariate logistic regression analysis indicated that age (OR   =  0.942, 95CI%  = 0.917–0.969, *P* < 0.001), surgical method (OR   =  0.318, 95CI%  = 0.171–0.592, *P* < 0.001), T2DM (OR   =  0.326, 95CI%  = 0.169–0.627, *P*   =  0.001), and hypertension (OR   =  0.430, 95CI%  = 0.238–0.777, *P*   =  0.005) were statistically associated with TWL% outcomes and included as confounders for further analysis (Table 2).

**Table 1:** Characteristics of the participants between the adequate group and inadequate group

Characteristic	Overall	Suboptimal clinical response group	Optimal clinical response group	*P*
Number	291	57	234	
Age		40.18±12.05	33.41±9.54	<0.001
BMI		36.10±9.37	40.35±8.39	0.001
Sex				0.873
Female	130	26	104	
Male	161	31	130	
T2DM				0.001
No	131	14	117	
Yes	160	43	117	
Surgery				<0.001
LSG	223	33	190	
RYGB	68	24	44	
Hypertension				0.005
No	166	23	143	
Yes	125	34	91	
Hyperlipidemia				0.117
No	86	12	74	
Yes	205	45	160	
Hyperuricemia				0.057
No	180	29	151	
Yes	111	28	83	

**Table 2:** Logistic analysis of predict factor of TWL% outcomes at one year after MBS

Characteristic	OR	95%CI	*P*
Age	0.942	0.917-0.969	<0.001
Sex			
Female	Ref		
Male	0.873	0.586-1.875	0.873
T2DM			
No	Ref		
Yes	0.326	0.169-0.627	0.001
Surgery			
LSG	Ref		
RYGB	0.318	0.171-0.592	<0.001
Hypertension			
No	Ref		
Yes	0.430	0.238-0.777	0.005
Hyperlipidemia			
No	Ref		
Yes	0.577	0.288-1.154	0.120
Hyperuricemia			
No	Ref		
Yes	0.569	0.317-1.021	0.059

### Logistic regression analysis of quartiles

**Fig. 1:**
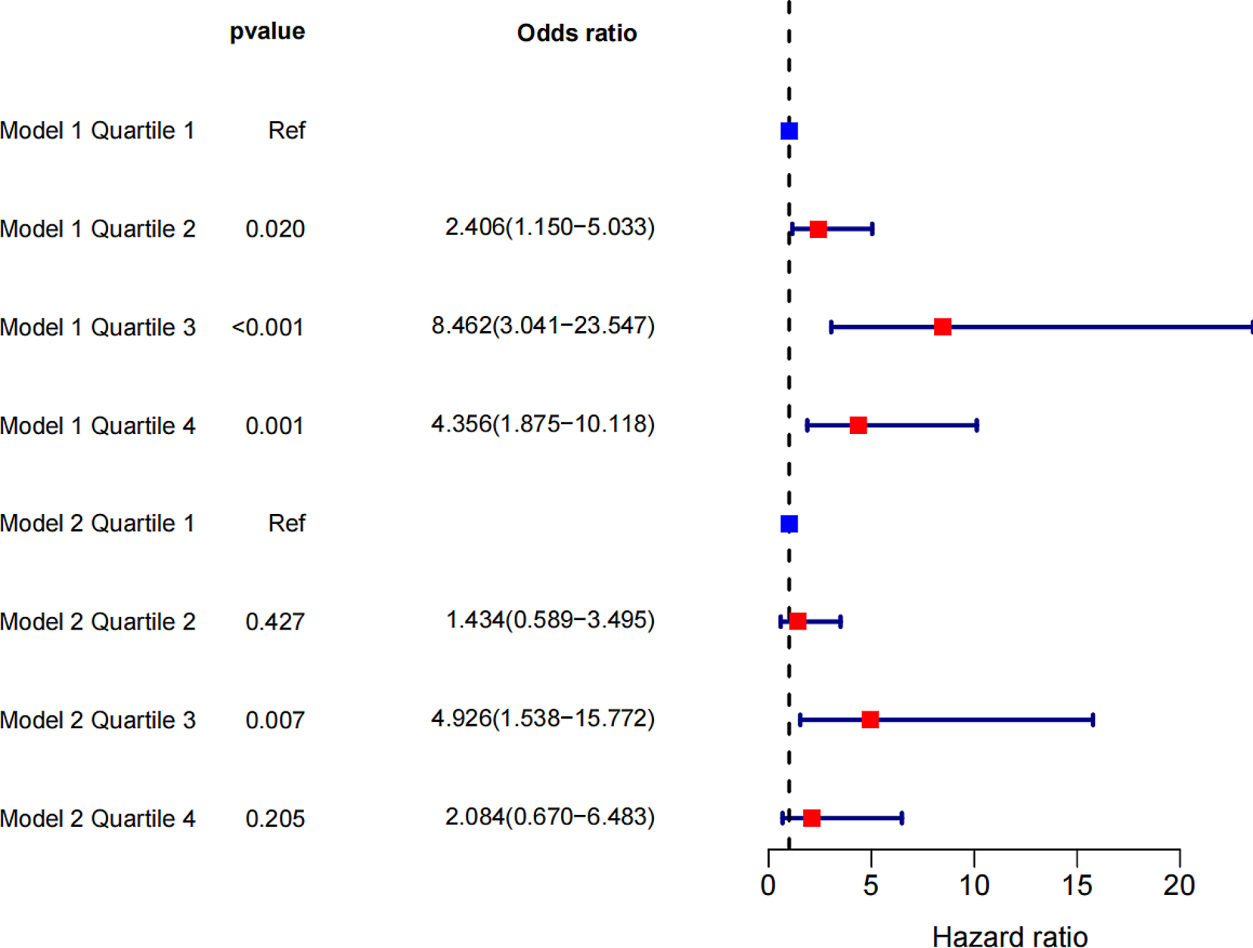
Odds ratios of logistic regression for BMI quartiles associated with optimal clinical response in models 1 and 2. The x axis shows odds ratios (ORs) of preoperative BMI associated with optimal clinical response. Data are shown as ORs with 95% confidence intervals. Model 1 was not adjusted. Model 2 was adjusted for potential confounders (age, surgical method, T2DM, and hyperuricemia). BMI: body mass index; T2DM: type 2 diabetes mellitus

The results of the logistic regression analysis of quartiles were shown in Fig. 1 and supplementary Table S2. The ORs (95% CI) for the preoperative BMI quartiles across low-to-high in Model 1 were 1.00 (reference), 2.406 (1.150–5.033), 8.462 (3.041–23.547), and 4.356 (1.875–10.118), respectively. In model 2, after adjusting for potential confounders, ORs (95% CI) for the preoperative BMI quartiles across low-to-high were 1.00 (reference), 1.434 (0.589–3.495), 4.926 (1.538–15.772), and 2.084 (0.670–6.483), respectively.

### Logistic regression analysis of ten groups according to per 2.5 kg/m^2^ units

**Fig. 2:**
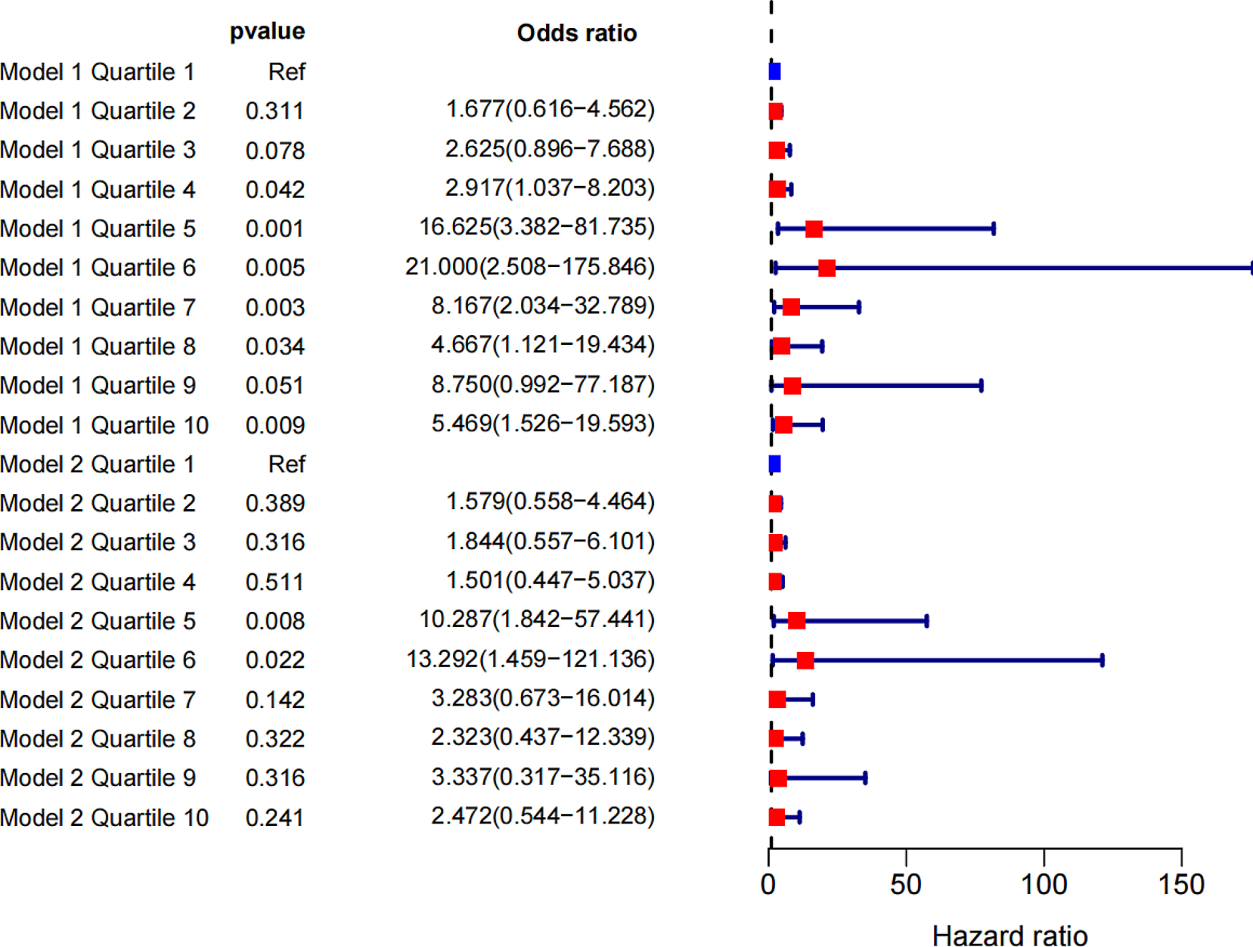
Odds ratios of logistic regression for per 2.5 kg/m^2^ units of BMI associated with optimal clinical response in models 1 and 2. The x axis shows odds ratios (ORs) of preoperative BMI associated with optimal clinical response. Data are shown as ORs with 95% confidence intervals. Model 1 was not adjusted. Model 2 was adjusted for potential confounders (age, surgical method, T2DM, and hyperuricemia). BMI: body mass index; T2DM: type 2 diabetes mellitus

The logistic regression analysis results of the ten groups according to per 2.5 kg/m^2^ units were shown in Fig. 2 and supplementary Table S3. In model 1, compared with the reference (27.5   ≤  BMI < 30.0), the ORs (95% CI) with statistical significance for optimal clinical response were 2.917 (1.037–8.203), 16.625 (3.382–81.735), 21.000 (2.508-175.846), 8.167 (2.034–32.789), 4.667 (1.121–19.434), and 5.469 (1.526–19.593) for BMI categories at 35.0   ≤  BMI < 37.5, 37.5   ≤  BMI < 40.0, 40.0   ≤  BMI < 42.5, 42.5   ≤  BMI < 45.0, 45.0   ≤  BMI < 47.5, and 50.0   ≤  BMI, respectively. In model 2, compared with the reference (27.5   ≤  BMI < 30.0), only 37.5   ≤  BMI < 40.0 [10.287 (1.842–57.441)] and 40.0   ≤  BMI < 42.5 [13.292 (1.459-121.136)] worked as predictive factors of TWL% outcome.

### Restricted cubic spline

**Fig. 3:**
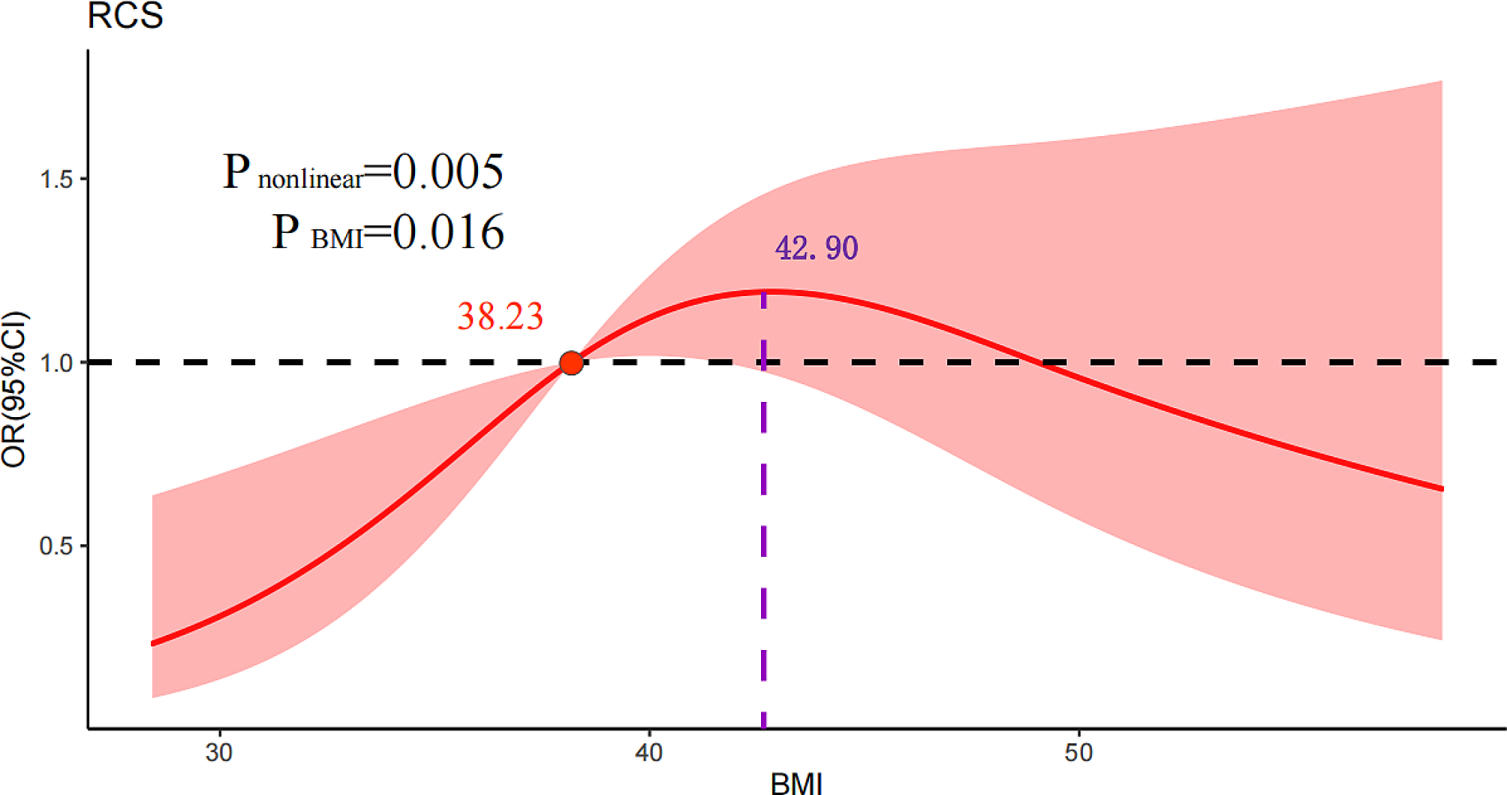
RCS curve of BMI and TWL% outcome in model 2. The y axis shows odds ratios (ORs) of preoperative BMI associated with optimal clinical response with the shaded area representing 95%CIs (nonlinear trend, P   =  0.005). Model was adjusted for potential confounders (age, surgical method, T2DM, and hyperuricemia). BMI: body mass index; T2DM: type 2 diabetes mellitus; TWL%: percentage of total weight loss; MBS: Metabolic and Bariatric Surgery: RCS: restricted cubic spline

The RCS method was used to fit a curve describing the association between preoperative BMI and optimal clinical response in model 1 (Supplementary Fig. S1) and model 2 (Fig. 3). RCS analysis showed a non-linear inverted U-shaped association between preoperative BMI and optimal clinical response (Nonlinear P   =  0.005). In spline analysis, when preoperative BMI was no less than 42.9 kg/m^2^, the possibility of optimal clinical response rose as preoperative BMI increased. When preoperative BMI was greater than 42.9 kg/m^2^, the possibility of optimal clinical response had a tendency to decline as BMI increased. The results of the subgroup RCS analyses based on surgery showed similar characteristics to the overall trend (Supplementary Fig. S2).

## Discussion

Several variables could be utilized to examine weight-loss outcomes after MBS, such as percent excess weight loss (EWL%), percent excess BMI loss (EBMIL%), and TWL% [28]. In our study, the TWL% outcome was chosen as the main observation indicator, allowing us to avoid distortion that could be generated by other indicators, such as EWL% and EBMIL% [29, 30]. Although numerous relevant studies have been performed, the effect of preoperative BMI on postoperative weight loss remains contentious. On the one hand, Al-Khyatt et al. suggested that preoperative factors for inadequate EWL% at 12 months after RYGB included higher initial BMI [31]. Not coincidentally, Livhits et al. concluded in a meta-analysis that preoperative pre-operative BMI might be negatively associated with weight loss [15]. Moreover, a similar opinion was proposed in a study by Ortega et al., who concluded that EWL% was negatively associated with BMI [32]. On the other hand, a study by Voglino et al. indicated that patients with TWL%≥20% had a lower preoperative BMI than those with TWL% < 20% [33]. Menenakos et al. also indicated that patients with BMI>50 kg/m^2^ achieved greater weight loss than those with BMI   ≤  50 kg/m^[2 [34]]^. Interestingly, Angrisani et al. came to a conclusion different from two perspectives above [35]. They found that the initial BMI was not associated with the EWL% at five years after the MBS. The differences in perspectives proposed by different studies may be attributable to factors such as the size of the sample, the various BMI categories, and the statistical methodology.

Our study tried to figure out the association between preoperative BMI and optimal clinical response from a nonlinear perspective. Using the RCS model with 3 nodes, our study discovered the nonlinear dose-response association between preoperative BMI and optimal clinical response. We identified an inverted U-shaped connection with an inflection point of the risk function at 42.9 kg/m^2^, and concluded on this premise that preoperative BMI was a predictive factor for optimal clinical response before 42.9 kg/m^2^, and a risk factor for optimal clinical response after 42.9 kg/m^2^. Increasing changes in preoperative BMI have an incremental or decremental influence on the expected value of TWL% outcomes and are linearly related in the conventional risk model [31, 32, 34]. However, according to the results of the RCS analysis, the possibility of optimal clinical response decreased in patients with severe obesity. To achieve optimal clinical response in patients with severe obesity, non-surgical methods such as intragastric balloon, etc. should be a pioneer first. Then, we can consider performed MBS, which leads to the optimal clinical response. In addition, for those patients with low-weight obesity, MBS could be performed under the premise of stricter indications, which could improve surgical outcomes.

In our research, we used two types of segmented logistic regression methods: one based on preoperative BMI quartiles (Q1, Q2, Q3, and Q4) for grouping, and the other dividing the data into 10 groups. Through logistic regression analysis, we observed that within each segment, the effects appeared to be standardized, with jumps occurring at the nodal positions, which is clearly contrary to reality. In contrast, RCS effectively solved this problem, providing a more realistic, continuous and smooth transition of the effects, avoiding the unnatural jumps at the node positions. To our knowledge, our research was the first retrospective study to assess the association between preoperative BMI and TWL% outcome from a non-linear perspective using RCS. An inverted U-shaped curve was observed between preoperative BMI and optimal clinical response. The closer the data fit is to the genuine shape of the association curve, the better the predictive performance will be. Specifically, before the peak risk point 42.9 kg/m^2^, the possibility of optimal clinical response rose as preoperative BMI increased. After that, the possibility of optimal clinical response decreased with a continued increase in preoperative BMI. Moreover, the participants in this study were patients with obesity from China. After adjusting for related confounding factors, a significant nonlinear dose-response relationship between BMI and the optimal clinical response continues to exist (Nonlinear P  =  0.005). Interestingly, the results of the subgroup RCS analyses showed similar characteristics to the overall trend, suggesting some consistency between the different surgical modalities and TWL% outcomes. Therefore, our research may provide Asian patients with customized advice according to their preoperative BMI, increasing their possibility of optimal clinical response.

2Despite the fact that this study provided an objective examination of the relationship between preoperative BMI and optimal clinical response from a nonlinear perspective, there were still some shortcomings. Firstly, the sample size of our study was relatively insufficient, and a multicenter prospective study is still needed for validation. Secondly, this study investigated the association between preoperative BMI and optimal clinical response at one year, and the relationship between preoperative BMI and long-term optimal clinical response remains to be investigated. However, there are still other elements that may influence the optimal clinical response, so a thorough evaluation of the patient remains essential.

## Conclusion

Our research indicated the non-linear inverted U-shaped correlation between preoperative BMI and adequate weight loss. Setting a preoperative BMI threshold of 42.9 is critical to predicting optimal clinical outcomes.

### Electronic supplementary material

Below is the link to the electronic supplementary material.

Supplementary Material 1

## Data Availability

All relevant data and information can be obtained from the corresponding author upon reasonable request.

## References

[1] Lin X, Li H, Obesity (2021). Epidemiology, pathophysiology, and Therapeutics[J]. Front Endocrinol.

[2] Ortega FB, Lavie CJ, Blair SN (2016). Obesity and Cardiovascular Disease[J]. Circul Res.

[3] Jaacks LM, Vandevijvere S, Pan A (2019). The obesity transition: stages of the global epidemic[J]. Lancet Diabetes Endocrinol.

[4] Swinburn BA, Sacks G, Hall KD (2011). The global obesity pandemic: shaped by global drivers and local environments[J]. Lancet.

[5] Fink J, Seifert G, Blüher M (2022). Obesity surgery—weight loss, metabolic changes, oncological effects, and follow-up[J]. Deutsches Ärzteblatt International.

[6] Milaneschi Y, Simmons WK, van Rossum EF, C (2019). Depression and obesity: evidence of shared biological mechanisms[J]. Mol Psychiatry.

[7] Kolb R, Sutterwala FS, Zhang W (2016). Obesity and cancer: inflammation bridges the two[J]. Curr Opin Pharmacol.

[8] Weihrauch-Bluher S, Schwarz P, Klusmann JH (2019). Childhood obesity: increased risk for cardiometabolic disease and cancer in adulthood[J]. Metabolism.

[9] Wadden TA, Webb VL, Moran CH (2012). Lifestyle Modif Obesity[J] Circulation.

[10] Curry SJ, Krist AH, Owens DK (2018). Behavioral weight loss interventions to prevent obesity-related morbidity and mortality in Adults[J]. JAMA.

[11] Alamuddin N, Bakizada Z, Wadden TA (2016). Management of Obesity[J]. J Clin Oncol.

[12] Cornejo-Pareja I, Clemente-Postigo M, Tinahones FJ (2019). Metabolic and endocrine consequences of bariatric Surgery[J]. Front Endocrinol (Lausanne).

[13] Chen G, Zhang G, Peng B (2021). Roux-En-Y gastric bypass Versus Sleeve Gastrectomy Plus procedures for treatment of morbid obesity: systematic review and Meta-Analysis[J]. Obes Surg.

[14] Varban OA, Cassidy RB, Bonham A (2017). Factors Associated with achieving a body Mass Index of Less Than 30 after bariatric Surgery[J]. JAMA Surg.

[15] Livhits M, Mercado C, Yermilov I (2012). Preoperative predictors of weight loss following bariatric surgery: systematic Review[J]. Obes Surg.

[16] Cooper TC, Simmons EB, Webb K (2015). Trends in Weight Regain following Roux-en-Y gastric bypass (RYGB) bariatric Surgery[J]. Obes Surg.

[17] Schutz Y, Pataky Z, Editorial (2022). Beyond bariatric surgery: expected and unexpected long-term Evolution[J]. Front Endocrinol.

[18] Adams ST, Salhab M, Hussain ZI (2013). Roux-en-Y gastric bypass for morbid obesity: what are the preoperative predictors of weight loss?[J]. Postgrad Med J.

[19] Romero-Corral A, Montori VM, Somers VK (2006). Association of bodyweight with total mortality and with cardiovascular events in coronary artery disease: a systematic review of cohort studies[J]. Lancet.

[20] Nickel F, de la Garza JR, Werthmann FS (2019). Predictors of risk and success of obesity Surgery[J]. Obes Facts.

[21] Masrur M, Bustos R, Sanchez-Johnsen L (2020). Factors Associated with weight loss after metabolic surgery in a multiethnic sample of 1012 Patients[J]. Obes Surg.

[22] Mohammed MR, Mahdy T, Hashem A (2021). Impact of baseline BMI and adherence to Follow-Up on the outcome of Sleeve Gastrectomy in treatment of adolescent Obesity[J]. Obes Surg.

[23] Sisik A, Basak F (2020). Presurgical predictive factors of excess weight loss after laparoscopic sleeve Gastrectomy[J]. Obes Surg.

[24] Lusa L, Ahlin Č (2020). Restricted cubic splines for modelling periodic data[J]. PLoS ONE.

[25] Desquilbet L, Mariotti F. Dose-response analyses using restricted cubic spline functions in public health research. Stat Med. 2010;29(9):n/a–n/a. 10.1002/sim.3841 20087875

[26] Li Z, Chen G, Sang Q (2023). A nomogram based on adipogenesis-related methylation sites in intraoperative visceral fat to predict EWL% at 1 year following laparoscopic sleeve gastrectomy. Surg Obes Relat Dis.

[27] Du D, Wang L, Chen W (2022). Weight loss at six months is the surrogate of long-term treatment outcomes for obese Chinese with a BMI less than 35 kg/m2 undergoing Roux-en-Y gastric bypass. Asian J Surg.

[28] van de Laar AW, van Rijswijk AS, Kakar H (2018). Sensitivity and specificity of 50% excess weight loss (50%EWL) and twelve other bariatric criteria for weight loss Success[J]. Obes Surg.

[29] van de Laar AW, J M (2014). Algorithm for weight loss after gastric bypass surgery considering body mass index, gender, and age from the bariatric outcome longitudinal database (BOLD)[J]. Surg Obes Relat Dis.

[30] van de Laar AW, Dollé MH, de Brauw LM (2015). Which Baseline Weight should be Preferred as reference for weight loss results? Insights in Bariatric Weight loss mechanisms by comparing primary and revision gastric bypass Patients[J]. Obes Surg.

[31] Al-Khyatt W, Ryall R, Leeder P (2017). Predictors of inadequate weight loss after laparoscopic gastric bypass for morbid Obesity[J]. Obes Surg.

[32] Ortega E, Morínigo R, Flores L (2012). Predictive factors of excess body weight loss 1 year after laparoscopic bariatric surgery[J]. Surg Endosc.

[33] Voglino C, Badalucco S, Tirone A (2022). Follow-up after bariatric surgery: is it time to tailor it? Analysis of early predictive factors of 3-year weight loss predictors of unsuccess in bariatric patients[J]. Updates Surg.

[34] Menenakos E, Stamou M, Albanopoulos K (2010). Laparoscopic sleeve gastrectomy performed with intent to treat morbid obesity: a prospective single-center study of 261 patients with a median follow-up of 1 Year[J]. Obes Surg.

[35] Angrisani L, Lorenzo ND, Favretti F (2004). The Italian Group for LAP-BAND: predictive value of initial body mass index for weight loss after 5 years of follow-up[J]. Surg Endosc.

