# Supplementary material for: Analysis of correlation between BMI and TWL% outcome following metabolic and bariatric surgery: a retrospective study using restricted cubic spline
Source: BMC Surg. 2024 Jun 7;24:178. doi: 10.1186/s12893-024-02455-7 (PMC11157919; doi:10.1186/s12893-024-02455-7)
Supplement: Supplementary file 1 — Supplementary Material 1 [file 12893_2024_2455_MOESM1_ESM.docx]

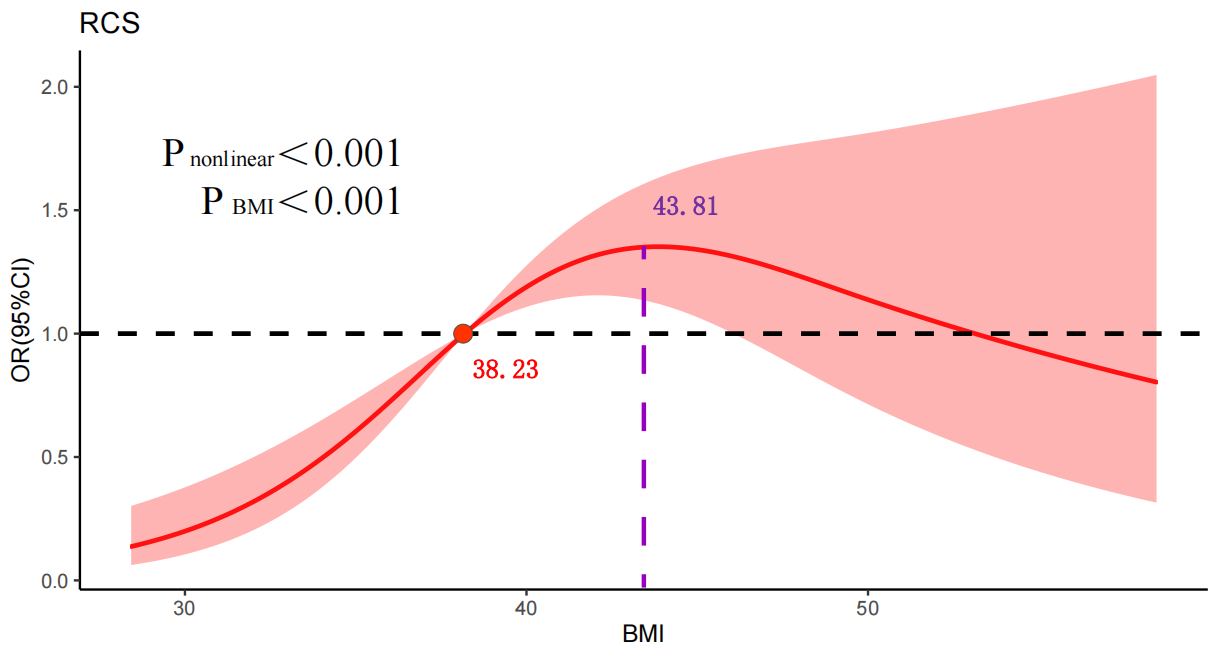


**Supplementary Figure 1. RCS curve of BMI and TWL% outcome in model 1.** The y axis shows odds ratios (ORs) of preoperative BMI associated with adequate the optimal clinical response with the shaded area representing 95%CIs (nonlinear trend, P <0.05). BMI: body mass index; T2DM: type 2 diabetes mellitus; TWL%: percentage of total weight loss; RCS: restricted cubic spline.


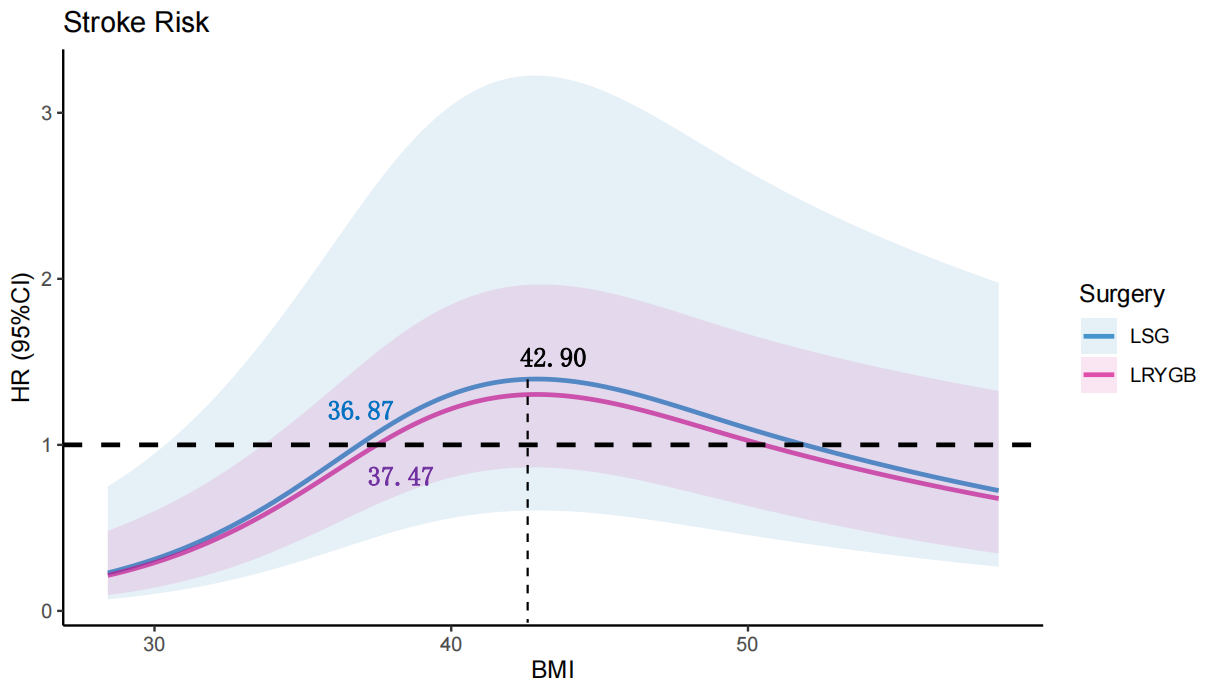


**Supplementary Figure 2. Subgroup RCS analysis based on surgery**

The y axis shows odds ratios (ORs) of preoperative BMI associated with the optimal clinical response with the shaded area representing 95%CIs for different surgeries. BMI: body mass index; LSG: laparoscopic sleeve gastrectomy; LRYGB: Laparoscopic Roux-en-Y gastric bypass.

| Supplementary table 1. Characteristics of the participants among quarlites | | | | | | |
| --- | --- | --- | --- | --- | --- | --- |
| Characteristic | Overall | Q1 | Q2 | Q3 | Q4 | P |
| Number | 291 | 69 | 77 | 70 | 75 | ＜0.001 |
| Age |  | 42.36±10.68 | 34.85±10.32 | 32.25±9.08 | 29.40±6.37 | ＜0.001 |
| BMI |  | 30.33±1.60 | 35.76±1.52 | 40.85±1.75 | 51.30±7.76 | ＜0.001 |
| Sex |  |  |  |  |  | ＜0.001 |
| Female | 130 | 34 | 18 | 32 | 46 |  |
| Male | 161 | 39 | 55 | 41 | 26 |  |
| T2DM |  |  |  |  |  | ＜0.001 |
| No | 131 | 1 | 38 | 38 | 54 |  |
| Yes | 160 | 72 | 35 | 35 | 18 |  |
| Surgery |  |  |  |  |  | ＜0.001 |
| LSG | 223 | 26 | 60 | 68 | 69 |  |
| RYGB | 68 | 47 | 13 | 5 | 3 |  |
| Hypertension |  |  |  |  |  | 0.449 |
| No | 166 | 38 | 46 | 44 | 38 |  |
| Yes | 125 | 35 | 27 | 29 | 34 |  |
| Hyperlipidemia |  |  |  |  |  | 0.036 |
| No | 86 | 14 | 22 | 30 | 20 |  |
| Yes | 205 | 59 | 51 | 43 | 52 |  |
| Hyperuricemia |  |  |  |  |  | 0.439 |
| No | 180 | 51 | 43 | 44 | 42 |  |
| Yes | 111 | 22 | 30 | 29 | 30 |  |

| Supplementary table 2. Logistic regression analysis of quartiles in model 1 and model 2 | | | |
| --- | --- | --- | --- |
| Characteristic | OR | 95%CI | P |
| Model 1 Quartile 1 (lowest) | Ref |  |  |
| Model 1 Quartile 2 | 2.406 | 1.150-5.033 | 0.02 |
| Model 1 Quartile 3 | 8.462 | 3.041-23.547 | ＜0.001 |
| Model 1 Quartile 4 (highest) | 4.356 | 1.875-10.118 | 0.001 |
| Model 2 Quartile 1 (lowest) | Ref |  |  |
| Model 2 Quartile 2 | 1.434 | 0.589-3.495 | 0.427 |
| Model 2 Quartile 3 | 4.926 | 1.538-15.772 | 0.007 |
| Model 2 Quartile 4 (highest) | 2.084 | 0.670-6.483 | 0.205 |
| Model 2 Age | 0.973 | 0.941-1.005 | 0.099 |
| Model 2 T2DM |  |  |  |
| No | Ref |  |  |
| Yes | 0.694 | 0.296-1.629 | 0.401 |
| Model 2 Surgery |  |  |  |
| LSG | Ref |  |  |
| RYGB | 0.783 | 0.351-1.745 | 0.549 |
| Model 2 Hypertension |  |  |  |
| No | Ref |  |  |
| Yes | 0.487 | 0.256-0.928 | 0.029 |

| Supplementary table 3. Logistic regression analysis of per 2.5kg/m2 units in model 1 and model 2 | | | |
| --- | --- | --- | --- |
| Characteristic | OR | 95%CI | P |
| Model 1 27.5≤BMI＜30.0 | Ref |  |  |
| Model 1 30.0≤BMI＜32.5 | 1.677 | 0.616-4.562 | 0.311 |
| Model 1 32.5≤BMI＜35.0 | 2.625 | 0.896-7.688 | 0.078 |
| Model 1 35.0≤BMI＜37.5 | 2.917 | 1.037-8.203 | 0.042 |
| Model 1 37.5≤BMI＜40.0 | 16.625 | 3.382-81.735 | 0.001 |
| Model 1 40.0≤BMI＜42.5 | 21.000 | 2.508-175.846 | 0.005 |
| Model 1 42.5≤BMI＜45.0 | 8.167 | 2.034-32.789 | 0.003 |
| Model 1 45.0≤BMI＜47.5 | 4.667 | 1.121-19.434 | 0.034 |
| Model 1 47.5≤BMI＜50.0 | 8.750 | 0.992-77.187 | 0.051 |
| Model 1 50.0≤BMI | 5.469 | 1.526-19.593 | 0.009 |
| Model 2 27.5≤BMI＜30.0 | Ref |  |  |
| Model 2 30.0≤BMI＜32.5 | 1.579 | 0.558-4.464 | 0.389 |
| Model 2 32.5≤BMI＜35.0 | 1.844 | 0.557-6.101 | 0.316 |
| Model 2 35.0≤BMI＜37.5 | 1.501 | 0.447-5.037 | 0.511 |
| Model 2 37.5≤BMI＜40.0 | 10.287 | 1.842-57.441 | 0.008 |
| Model 2 40.0≤BMI＜42.5 | 13.292 | 1.459-121.136 | 0.022 |
| Model 2 42.5≤BMI＜45.0 | 3.283 | 0.673-16.014 | 0.142 |
| Model 2 45.0≤BMI＜47.5 | 2.323 | 0.437-12.339 | 0.322 |
| Model 2 47.5≤BMI＜50.0 | 3.337 | 0.317-35.116 | 0.316 |
| Model 2 50.0≤BMI | 2.472 | 0.544-11.228 | 0.241 |
| Model 2 Age | 0.967 | 0.935-1.001 | 0.056 |
| Model 2 T2DM |  |  |  |
| No | Ref |  |  |
| Yes | 0.669 | 0.281-1.589 | 0.362 |
| Model 2 Surgery |  |  |  |
| LSG | Ref |  |  |
| RYGB | 0.883 | 0.382-2.039 | 0.770 |
| Model 2 Hypertension |  |  |  |
| No | Ref |  |  |
| Yes | 0.507 | 0.264-0.974 | 0.041 |
